# BET Bromodomain Inhibitors Which Permit Treg Function Enable a Combinatorial Strategy to Suppress GVHD in Pre-clinical Allogeneic HSCT

**DOI:** 10.3389/fimmu.2018.03104

**Published:** 2019-01-24

**Authors:** Sabrina N. Copsel, Casey O. Lightbourn, Henry Barreras, Ines Lohse, Dietlinde Wolf, Cameron S. Bader, John Manov, Brandon J. Kale, Devangi Shah, Shaun P. Brothers, Victor L. Perez, Krishna V. Komanduri, Claes Wahlestedt, Robert B. Levy

**Affiliations:** ^1^Department of Microbiology and Immunology, Miller School of Medicine, University of Miami, Miami, FL, United States; ^2^Sylvester Comprehensive Cancer Center, Miller School of Medicine, University of Miami, Miami, FL, United States; ^3^Center for Therapeutic Innovation and Department of Psychiatry and Behavior Sciences, Miller School of Medicine, University of Miami, Miami, FL, United States; ^4^Department of Ophthalmology, Miller School of Medicine, University of Miami, Miami, FL, United States; ^5^Department of Medicine, Miller School of Medicine, University of Miami, Miami, FL, United States

**Keywords:** Tregs, bromodomain inhibitors, epigenetic regulation, GVHD, TNFRSF25, CD25

## Abstract

A recent approach for limiting production of pro-inflammatory cytokines has been to target bromodomain and extra-terminal (BET) proteins. These epigenetic readers of histone acetylation regulate transcription of genes involved in inflammation, cardiovascular disease, and cancer. Development of BET inhibitors (BETi) has generated enormous interest for their therapeutic potential. Because inflammatory signals and donor T cells promote graft-versus-host disease (GVHD), regulating both pathways could be effective to abrogate this disorder. The objective of the present study was to identify a BETi which did not interfere *in vivo* with CD4^+^FoxP3^+^ regulatory T cell (Treg) expansion and function to utilize together with Tregs following allogeneic hematopoietic stem cell transplantation (aHSCT) to ameliorate GVHD. We have reported that Tregs can be markedly expanded and selectively activated with increased functional capacity by targeting TNFRSF25 and CD25 with TL1A-Ig and low dose IL-2, respectively. Here, mice were treated over 7 days (TL1A-Ig + IL-2) together with BETi. We found that the BETi EP11313 did not decrease frequency/numbers or phenotype of expanded Tregs as well as effector molecules, such as IL-10 and TGF-β. However, BETi JQ1 interfered with Treg expansion and altered subset distribution and phenotype. Notably, in Treg expanded mice, EP11313 diminished tnfa and ifng but not il-2 RNA levels. Remarkably, Treg pSTAT5 expression was not affected by EP11313 supporting the notion that Treg IL-2 signaling remained intact. MHC-mismatched aHSCT (B6 → BALB/c) was performed using *in vivo* expanded donor Tregs with or without EP11313 short-term treatment in the recipient. Early post-transplant, improvement in the splenic and LN CD4/CD8 ratio along with fewer effector cells and high Treg levels in aHSCT recipients treated with expanded Tregs + EP11313 was detected. Interestingly, this group exhibited a significant diminution of GVHD clinical score with less skin and ocular involvement. Finally, using low numbers of highly purified expanded Tregs, improved clinical GVHD scores were observed in EP11313 treated recipients. In total, we conclude that use of this novel combinatorial strategy can suppress pre-clinical GVHD and posit, *in vivo* EP11313 treatment might be useful combined with Treg expansion therapy for treatment of diseases involving inflammatory responses.

## Introduction

Allogeneic hematopoietic stem cell transplantation (aHSCT) is utilized as a therapeutic modality for a variety of conditions including genetic disorders, immune deficiency syndromes, and hematologic diseases and malignancies. However, the limiting factor for successful aHSCT is the development of graft-versus-host disease (GVHD). In fact, as many as half of the ~8,000 aHSCTs performed in the U.S. each year will result in GVHD and therefore new strategies to ameliorate GVHD are needed. GVHD occurs when donor T cells are primed by recipient antigens subsequently eliciting an inflammatory response against the host (1). Acute GVHD is a multi-organ disorder resulting from inflammatory cytokines and donor T cells which primarily damage skin, liver, gastrointestinal tract, and the eye surface (2).

Because GVHD is promoted by donor T cells and inflammatory cytokines, we reason *regulating both* is the most rational strategy to abrogate this complication. Our lab and others have demonstrated that transfer of CD4^+^FoxP3^+^ regulatory T cells (Tregs) is a promising therapy to suppress donor T cells and inhibit GVHD (3–6). Our prior work identified a two-pathway *in vivo* strategy targeting TNFRSF25 and CD25 receptors which elicits a rapid and strong increase in Treg numbers and function (7). In fact, very low numbers of these *in vivo* expanded donor Treg cells demonstrated effective GVHD suppression in recipients following aHSCT (8). Recently, the targeting of bromodomain and extra-terminal (BET) proteins has provided a new strategy for reducing pro-inflammatory cytokine production (9). These readers of histone acetyled lysine residues are involved in transcriptional regulation of many genes involved in human diseases including inflammation, cancer and cardiovascular diseases (10, 11). Recent development of BET inhibitors (BETi) has generated enormous interest for their therapeutic potential (12–14). The BETi I-BET762 and JQ1 showed anti-inflammatory properties by disrupting the expression of pro-inflammatory cytokines (e.g., IL-1β, IL-6, and IL-12) in macrophages and suppressing genes involved in T cell-mediated pro-inflammatory functions (13, 15, 16). A prior study reported that BETi I-BET151 interfered with NF-κb function and diminished cytokine expression in dendritic cells and T cells, altered APC function and decreased experimental GVHD (17). Based on our previous work illustrating the effectiveness of expanded Tregs in ameliorating GVHD, we wanted to ask if BETi could be combined with this cell therapy to augment outcomes of aHSCT. Small biomolecule inhibition of CBP/EP300 bromodomains resulted in diminishment of Treg frequency and differentiation (18). It is notable that STAT5 activation is required for Treg proliferation and function (19, 20). Importantly, although JQ1 was shown to reduce STAT5 function in hematologic cancers and dendritic cells, there is no information regarding this or other BETi effects on (1) the IL-2 signaling pathway via STAT5 in Tregs as well as (2) IL-2 production which is required for Treg survival and their maintenance of suppressive function (21, 22).

The present studies examined if BETi could be combined with Treg cell therapy without interfering with Treg expansion, phenotype and function. We found that the BETi EP11313 did not decrease Treg numbers in treated mice and in Treg expanded mice, EP11313 diminished tnfa and ifng but not il-2 levels in non-Treg cells. Notably, Treg pSTAT5 expression was not affected by EP11313 supporting the notion that Treg IL-2 signaling remained intact. In the presence of this BETi, no alterations in Treg subsets or phenotype markers as well as effector molecules, such as IL-10 and TGF-β were observed. MHC-mismatched aHSCT (donor B6-BALB/c recipient) was performed using *in vivo* expanded donor Tregs with or without EP11313 treatment in the recipient. One week post-transplant we observed significant improvement in the splenic and LN CD4/CD8 ratio along with fewer effector cells and high Treg levels in HSCT recipients treated with expanded Tregs + EP11313. Remarkably, this group exhibited diminished acute GVHD. Finally, using low numbers of highly purified expanded Tregs, we found improved clinical GVHD scores in recipients treated with EP11313. We conclude *in vivo* treatment with selective BETi can be successfully combined with Treg expansion therapy for treatment of diseases involving inflammatory responses.

## Materials and Methods

### Mice and Reagents

The FoxP3 reporter mice on a C57BL/6 background (B6-FoxP3^RFP^) (originally provided by R. Flavell, Yale University, New Haven, CT) (23) and B6-CD45.1 (H2^b^) mice were bred in our facility. Wild-type BALB/c (H2^d^) mice were purchased from Taconic (Rensselaer, NY). Mice were used at 6–12 weeks of age and were maintained in pathogen-free conditions at the University of Miami animal facilities. All animal use procedures were approved by the University of Miami Institutional Animal Care and Use Committee. EP11313 (provided by Neomed, Canada) and JQ1 (kindly provided by Dr. James Bradner) were reconstituted in DMSO and further diluted in Tween 80 and saline. The A20^luc/YFP^ cell line (derived from BALB/c mice) was a generous gift of Dr. Robert Negrin (Stanford University).

### Antibodies, Reagents, Flow Cytometry, and Cell Sorting

Commercial antibodies for use in flow cytometry were purchased from BD Biosciences (San Jose, CA), Biolegend (San Diego, CA), or eBioscience (Waltham, MA). Recombinant mouse IL-2 and α-IL-2 monoclonal antibody, clone JES6-5H4, were purchased from eBioscience. IL-2/αIL-2 complex was generated by incubating 1.5/7.5 μg recombinant mouse IL-2 with 8μg JES6-5H4 (~8,000 IU/injection) for 15 min at room temperature and administered i.p. TL1A-Ig was generated in our laboratory as described previously (24) and administered i.p. at 50 μg/mouse/injection. Single-cell suspensions were prepared from different organs (spleen and lymph nodes). Peripheral blood was collected in heparinized tubes. Peripheral blood mononuclear cells were isolated by standard Ficoll density gradient centrifugation. Next, 10^6^ cells were preblocked with anti-mouse CD16/CD32 and stained with different antibody combinations. Intracellular staining was performed according to standard procedures. The following mAbs to the indicated molecules, the fluorescent labels, and their sources were used in this study: CD4, CD8, CD19, CD25, CD44, CD62L, CD103, KLRG1, CD39, CD73, I-COS, Nrp-1, PD-1, CTLA-4, CCR8, Ly-6C, Ki-67, pSTAT5, and Annexin V (Supplementary Materials and Methods, Table S1). Flow cytometric analysis was performed on a BD LSR-Fortessa-HTS instrument (BD Biosciences, San Jose, CA) and the analysis was completed using FlowJo software (FlowJo, LLC, Ashland, OR). Splenic and pLN CD4^−^FoxP3^−^, CD4^+^FoxP3^−^, and CD4^+^FoxP3^+^ cells were sorted using a FACS Aria II cell sorter (BD Biosciences) after enrichment of T cells (surface immunoglobulin depletion of B cells).

### RNA Isolation, RT-PCR, and Quantitative Real-Time PCR

Total RNA was isolated from unexpanded and expanded Tregs using RNAeasy mini kit following the manufacturer's instructions (Qiagen, Germantown, MD). cDNA was retrotranscribed from 1 μg of total RNA using qScript cDNA Mastermix (Quanta, Beverly, MA). Quantitative real-time PCR was (qPCR) was performed in triplicate using the ABI PRISM 7300 sequence detection system (Applied Biosystems, Whatman, MA) with the specific primers for tnfa, ifng, il-2, il-10, and gapdh (Supplementary Materials and Methods, Table S2). The PCR mixture contained 7.5 μl of 2X SYBR Green PCR master mix (Applied Biosystems) in a 15 μl final volume. The specificity of each primer set was monitored by analyzing the dissociation curve. The relative mRNA levels of each gene were calculated using the Livak method with GAPDH as the housekeeping gene.

### Western Blot

Cells (0.5–1.0 × 10^6^) were diluted with Laemmli sample buffer and boiled for 5 min. Proteins were separated by electrophoresis on 4–15% SDS-polyacrylamide gel and transferred to nitrocellulose membranes. The residual binding sites were blocked with 5% non-fat powdered milk in PBS containing 0.05% Tween 20, and membranes were incubated with anti-TGF-b mAb (0.5 mg/ml; ABCAM, Cambridge, United Kingdom) or anti-actin mAb (0.5 mg/ml; ABCAM) in PBS containing 0.05% Tween 20. All subsequent washes were performed with the same buffer. Reactivity was developed using HRP-coupled secondary polyclonal antibody (1:2,000, Jackson ImmunoResearch) and the SuperSignal West Pico chemiluminescent substrate (Thermo Scientific).

### *In vitro* Treg Functional Assays

For a standard suppressor assay, CD4^+^FoxP3^−^ splenocytes (10^5^) were cultured in 96-well plates and activated with 1 μg soluble anti-CD3 (clone 2C11) antibody in the presence of APCs (5 × 10^4^ T cell depleted splenocytes) and titrating numbers of sorted CD4^+^FoxP3^+^ Tregs. Cultures were incubated for 72 h and pulsed with [^3^H]-Thymidine (0.5 μCi/well; Perkin Elmer) for the last 10 h. Incorporated isotope was measured by liquid scintillation counting (Micro Beta TriLux counter; Perkin Elmer). For a functional assay, spleen and LN suspensions were activated *in vitro* with 1 μg/ml soluble anti-CD3. After 72 h, proliferating cells were counted by Trypan blue exclusion using the Vi-cell XR cell counter (Beckman Coulter, Brea, CA).

### HSCT Experiments

For the HSCT in the major MHC-mismatch model (B6 → BALB/c), female BALB/c mice (H2^d^) were ablatively conditioned with 8.5 Gy total body irradiation 1 day before transplantation. BM cells were obtained from femurs, tibias, and vertebrae from sex-matched B6-CD45.1 (H2^b^; Thy1.2) donor animals. A single-cell suspension of marrow cells was prepared by flushing bones with a 21-gauge needle and the cells were filtered through a 100 μm nylon mesh. Donor marrow cells were depleted of T cells via complement mediated lysis using anti–T-cell–specific antibody HO-13-4 (hybridoma supernatant, mouse anti-Thy1.2 IgM; ATCC, Manassas, VA) generously provided by Dr. Bruce Blazar (University of Minnesota), anti-CD4 mAb (clone 72.4), anti-CD8mAb (clone H02.2), and rabbit complement (Cedarlane Laboratories, Burlington, Ontario, Canada). The marrow cells were incubated at 37°C for 45 min, washed twice in RPMI, and resuspended for HSCT. Marrow T cell depletion was routinely >99%. Donor T cells were prepared from spleens or LN obtained from C57BL/6-FoxP3^RFP^-expanded or unexpanded animals. Donor cells were stained for T cells (anti-CD4, clone RM4-5; anti CD8, clone 53-6-7) and adjusted to 1.0 × 10^6^ T cells per mouse before mixing with BM. Recipient mice were injected twice a day with EP11313 10 mg/kg (from day −2 to 4) and underwent transplantation (day 0) with T cell–depleted (TCD) BM (5.5 × 10^6^) and 1.0 × 10^6^ T cells i.v. in a 0.2 ml volume via tail vein injection. GVHD was assessed by monitoring recipients for changes in total body weight, clinical signs, and overall survival. The clinical signs of GVHD were recorded for individual mice. Recipients were scored on a scale from 0 to 2 for 5 clinical parameters (25): weight loss, diarrhea, fur texture, posture, and alopecia and for ocular lid score on a scale from 0 to 4 (26).

### Histologic Analysis

Briefly, tissues from animals 4–5 weeks after aHSCT were fixed in 10% formalin and embedded in paraffin. Sections were stained with hematoxylin-eosin (H&E) and images were acquired using the Keyence BZ-X700 microscope (Itasca, IL). Slides were scored following a modified system described by Kaplan et al. (27). In brief, 3–4 parameters were used to compare pathology scores between groups in the skin and the colon.

### Cell Survival Assay

Cells were seeded in 384-well micro-titer plates and incubated in a humidified environment at 37°C and 5% CO_2_ and cultured for 24 h, followed by incubation with the EP11313, JQ1, iBET762, and iBET151. Individual drugs will be dissolved in 100% DMSO and tested in triplicates starting at a nominal test concentration of 10 μM over a 20,000-fold concentration range to generate dose response curves allowing for calculation of half-maximal and maximal response concentrations. Cell viability was measured by bioluminescence after 72 h of drug exposure and dose response curves were generated for each compound.

### Cell Proliferation Assay

In order to monitor cell proliferation in response to treatment with EP11313, cells were seeded in 24-well plates (Corning, NY) at 10^4^ cells per well. Cells were treated with 0, 0.05, 0.1, 0.5, 1, and 1.5 μM EP11313 (*n* = 4 per treatment) and proliferation was evaluated using the Incucyte Zoom instrument (Essenbioscience, MI).

### Determination of Serum TNF-α Levels

Serum was collected from animals treated *in vivo* with LPS 1 mg/kg and EP11313 10 or 30 mg/kg. TNF-α concentration was determined by Mouse TNF-α ELISA MAX (Biolegend, San Diego, CA), following manufacturer's protocol. Analysis was performed using Benchmark Plus microplate spectrophotometer at 405 OD.

### Statistical Analysis

All graphing and statistical analysis were performed using GraphPad Prism (San Diego, CA). Values shown in graphs represent the mean of each group ± SEM. Survival data were analyzed with the Mantel-Cox log-rank test. Non-parametric unpaired two-tailed *t*-test was used for comparisons between two experimental groups, and multiple variable analysis was performed using ANOVA. A *P*-value < 0.05 was considered significant. Brackets identifying the groups being compared are presented in each figure where appropriate accompanied by the level of significance or absence of significance (ns).

## Results

### A Bromodomain Inhibitor (BETi)—EP11313 Does Not Interfere With Treg Expansion, Subsets, and *in vitro* Function

**Figure 1:**
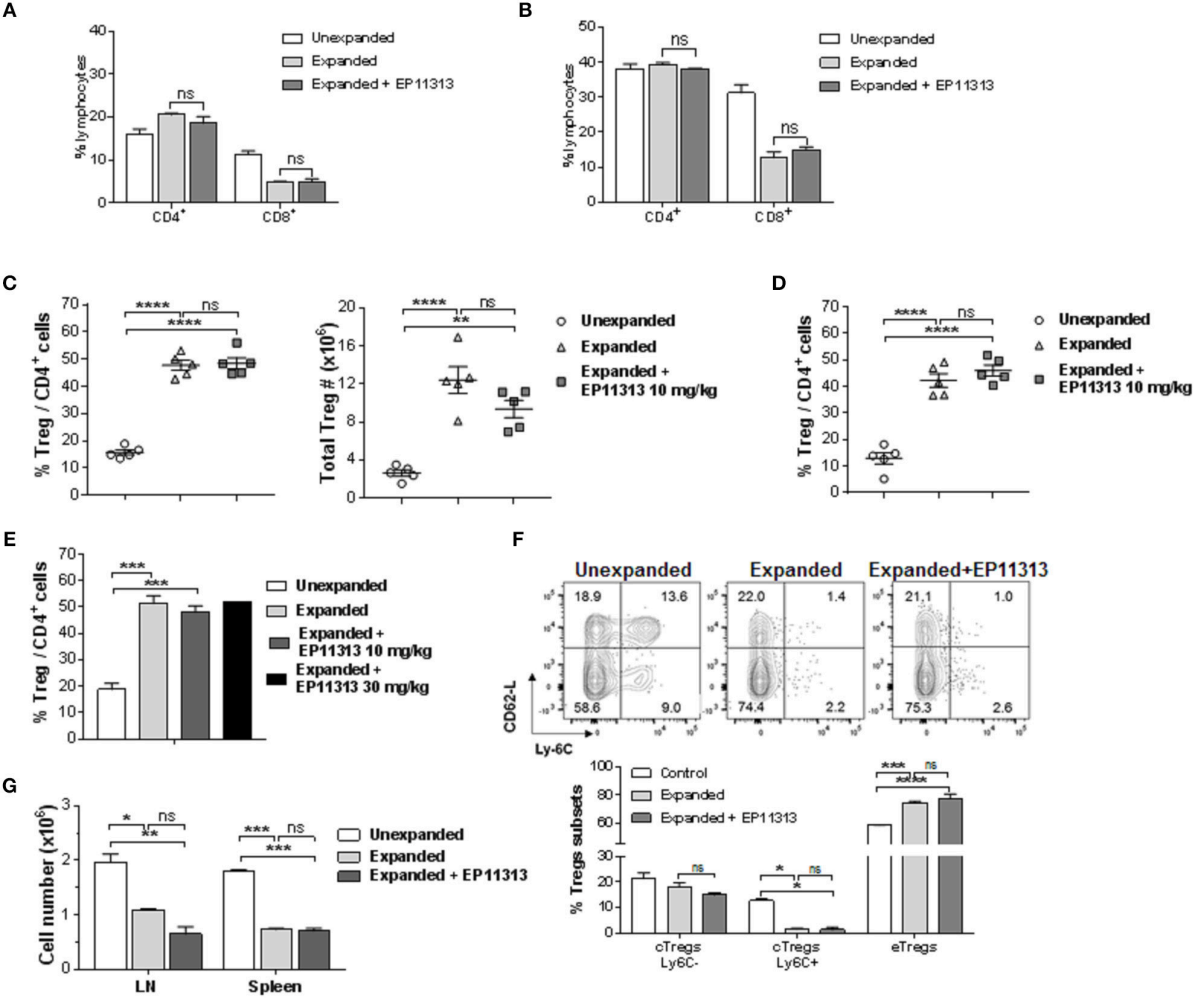
*In vivo* treatment with BETi EP11313 does not interfere with Treg expansion, subset distribution and *in vitro* suppressor function. **(A–G)** Mice were injected i.p. with TL1A-Ig (on days 1–4) and rmIL-2 bound to the anti-IL-2 mAb (JES6-5H4, on days 4 and 6) and EP11313 (10 mg/kg per dose) or administered vehicle on days −1 to 6 (twice per day). Mice were sacrificed on day 7. CD4^+^ or CD8^+^ frequency (%) in the spleen **(A)** and peripheral lymph nodes (pLN) **(B)** of mice undergoing Treg expansion (TL1A-Ig + low dose IL-2) in the presence or absence of BETi EP11313. Data representative of three independent experiments (*n* = 2 mice/group). Splenic overall Treg frequency (%) within the CD4 fraction (CD4^+^FoxP3^+^/CD4^+^) cells (left) and total numbers splenic Tregs (right) are shown **(C)**. Treg (CD4^+^FoxP3^+^) frequency (%) of total CD4^+^ cells in pLN **(D)**. Data are pooled from three independent experiments; *n* = 5 mice/group **(C,D)**. Treg frequency (%) of total CD4^+^ cells in expanded mice treated with EP11313 at 10 or 30 mg/kg **(E)**. Treg subset distribution determined by CD62-L and Ly-6C staining is shown as a representative contour plot (top) and a bar graph of data pooled from two independent experiments (bottom) **(F)**. No significant differences were observed in cTregs CD62L^hi^Ly-6C^−or+^ and eTregs CD62L^lo^Ly-6C^−^ in the Expanded + EP11313 treated mice vs. Expanded mice **(F)**. Treg expansion leads to a suppressive environment in spleen and LN which is not altered in the presence of BETi EP11313 **(G)**. Cell suspensions of spleen or lymph node cells obtained from indicated mice which underwent expansion treated with EP11313 (or vehicle) or normal, unexpanded mice. The cultures were then stimulated with anti-CD3mAb for 72 h **(G)**. Data are representative of two independent experiments. ns = not significant vs. expanded.*p < 0.05; **p < 0.01; ***p < 0.001; ****p < 0.0001 vs. unexpanded.

To determine if Treg exposure to BETi *in vivo* impaired homeostatic proliferation of CD4^+^FoxP3^+^ Tregs, C57BL/6-FoxP3^RFP^ (B6-FoxP3^RFP^) mice were administered EP11313 i.p. 2×/day (13 total injections) (Figure S1A in Supplementary Material). Spleen and lymph node (LN) cell analysis revealed no differences in the frequency of both the CD4^+^ compartment and Tregs (Figures S1B,C in Supplementary Material). Following Treg isolation via cell sorting (purity > 98%, d*ata not shown*) their *in vitro* functional activity was also not diminished as evidenced by a standard suppressor assay (Figure S1D in Supplementary Material). Since we employ expanded and proliferating Tregs for use to regulate GVHD (7, 8), we next addressed whether EP11313 altered expansion, subset distribution and function of Tregs undergoing two-pathway *in vivo* expansion following treatment with TL1A-Ig and low dose IL-2. Examination of peripheral (spleen, LN) lymphocytes indicated no change in the relative frequencies of CD4 and CD8 T cells in mice receiving two-pathway stimulation alone or together with EP11313 (Figures 1A,B, S1E in Supplementary Material). Importantly, the frequency and numbers of Treg cells within these two groups were also not altered (Figures 1C,D, S1F in Supplementary Material). Notably, following a 3× increase in EP11313 dose administration the percentage of Tregs within the CD4^+^ compartment was again not affected (Figure 1E). Utilizing Ly6C and CD62-L staining (28, 29), Treg subset evaluation demonstrated no change in the central Tregs (Ly6C^−^, CD62L^hi^ = cTregs) and effector Tregs (Ly6C^−^, CD62L^lo^ = eTregs) frequency between animals which had Tregs expanded in the presence or absence of the BETi. We previously found a diminution in the Ly6C^+^ Treg fraction following two-pathway expansion (8). Here, we also observed the same diminution in this subset in the presence or absence of EP11313 (Figure 1F). Lastly, to directly assess the functional activity of the Tregs expanded in the presence and absence of EP11313, spleen and LN cells were removed and immediately stimulated *in vitro* with anti-CD3mAb. The decrease in proliferation by cells from animals following Treg expansion was not significantly different regardless of whether animals also received BETi treatment (equivalent Treg suppressive activity) (Figure 1G).

It is well established that BETi possess anti-tumor activity (14). Therefore, to demonstrate activity of the BETs, we assessed tumor cell viability and proliferation using a mouse lymphoma cell line. As anticipated, each BETi examined decreased tumor cell viability and numbers at varying concentrations (Figure S2 in Supplementary Material). Since BETi are also known to inhibit transcription of inflammatory genes, we examined *in vivo* activity by the BETi EP11313. Following injection of two doses of this BETi, LPS was administered and TNF-α serum levels assessed after 1.5 h (Figure S3A in Supplementary Material). There was a clear decrease which was dose related in the serum levels of this inflammatory cytokine (Figure S3B in Supplementary Material).

### *In vivo* Administration of the JQ1 Decreases Treg Proliferation and Alters Their Phenotype During Expansion of CD4^+^FoxP3^+^ T Cells

**Figure 2:**
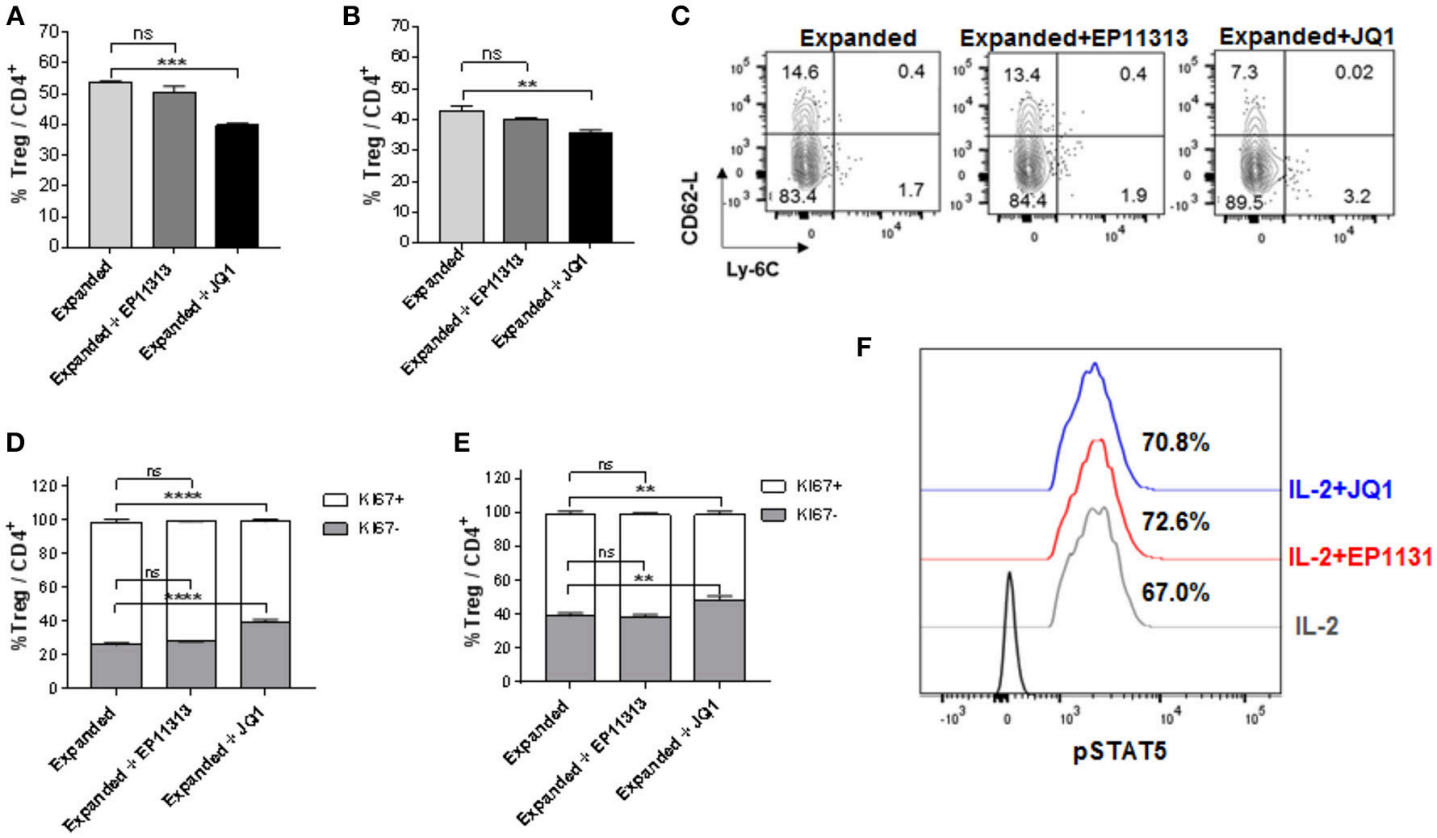
In contrast to BETi EP11313, JQ1 alters Treg frequency, proliferation and subsets with no differences in pSTAT5 expression. **(A–F)** Mice were injected i.p. with TL1A-Ig and rmIL-2 bound to anti-IL-2 mAb as in Figure 1 and EP11313 (10 mg/kg), JQ1 (5–10 mg/kg) or vehicle (on days −1 to 6). Mice were sacrificed on day 7. *in vivo* treatment with JQ1 significantly decreased overall Treg (CD4^+^FoxP3^+^) frequency (%) of total CD4^+^ cells in the spleen **(A)** and pLN **(B)**. Representative contour plot of Treg subset distribution determined by CD62L and Ly-6C staining of pLN from mice undergoing Treg expansion treated with EP11313, JQ1, or vehicle. JQ1 treatment diminished cTreg CD62L^hi^Ly6C^−^ **(C)**. Treg expanded proliferation was impaired with JQ1 *in vivo* treatment indicated by Ki67 expression in the spleen **(D)** and pLN **(E)**. **(A–E)** All results are representative of two independent experiments *n* = 3 mice/group. Representative histograms of lymph node cells from TL1A-Ig + IL-2 expanded mice treated *in vitro* with 500 nM BETi or vehicle and stimulated with IL-2 10 ng/ml for 15 min **(F)**. ns, not significant. **p < 0.01; ***p < 0.001; ****p < 0.0001 vs. expanded.

Next, we wanted to address if a prototypic BETi, JQ1 (12) exhibited the same pattern as EP11313 with regard to affecting Treg frequency, proliferation and subset distribution in Tregs undergoing expansion. Groups of B6-FoxP3^RFP^ mice were treated 2×/day (13 total injections, Figure S1E in Supplementary Material) with 5 mg/kg of JQ1 or 10 mg/kg of EP11313. In contrast to what we observed with EP11313 treatment, exposure of expanding Tregs to JQ1 resulted in a decrease in splenic and LN Treg frequencies (Figures 2A,B). A representative dot plot of Treg subsets showed that the cTregs (Ly6C^−^, CD62L^hi^) fraction was decreased in animals receiving JQ1—but not EP11313—in animals undergoing two-pathway Treg expansion (Figure 2C). Following Ki67 staining to assess cell proliferation, we observed that in contrast to EP11313, JQ1 treatment deceased the Ki67^+^ population within splenic and LN Tregs (Figures 2D,E). There was no effect of these BETi's on proliferation of conventional CD4^+^FoxP3^−^ T cells (Figure S4 in Supplementary Material). Lastly, IL-2 induced STAT5 phosphorylation of Tregs *in vitro* was not diminished in the presence of JQ1 or EP11313 (500 nM) (Figure 2F).

**Figure 3:**
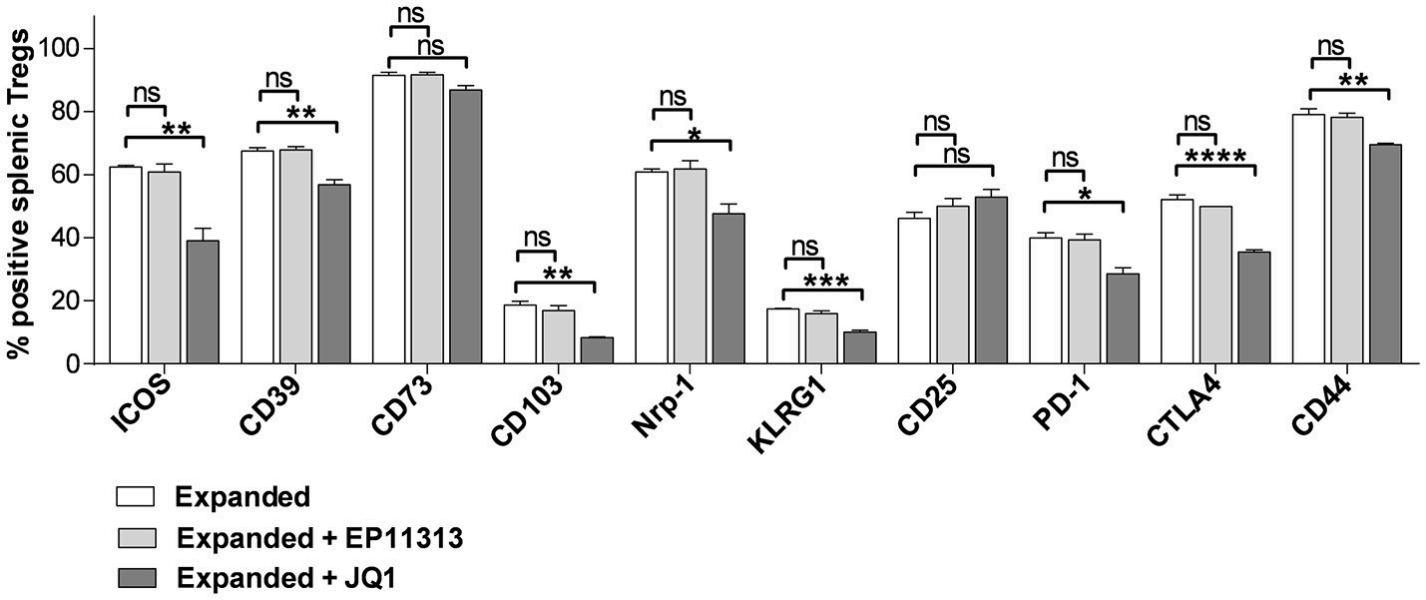
In contrast to BETi EP11313, JQ1 modifies Treg phenotype. Mice were injected i.p. with TL1A-Ig and rmIL-2 bound to anti-IL-2 mAb as in Figure 1 and EP11313 (10 mg/kg), JQ1 (5–10 mg/kg) or vehicle on days −1 to 6. Mice were sacrificed on day 7. Expression of activation, differentiation (i.e., ICOS, CD103, CD44, KLRG1) and functional (i.e., CD39, CD73, Nrp1, CTLA-4) molecules in splenic Tregs are shown. Data representative of five experiments (Treg expanded) and two independent experiments (expanded plus BETi) *n* = 3 mice/group. *p < 0.05; **p < 0.01; ***p < 0.001; ****p < 0.0001 vs. expanded.

To more precisely analyze expanded Treg phenotype in the presence of BETi treatment, activation, function and differentiation markers were assessed using mAbs to defined Treg proteins (Table S1 in Supplementary Materials and Methods). Notably, significantly decreased levels of activation and differentiation molecules, specifically ICOS, CD103, PD-1, CD44, and KLRG1 were identified in splenic Tregs undergoing expansion treated with 10 mg/kg of JQ1 (Figure 3). Additionally, the Treg functional suppressive mediators CD39, Nrp-1, and CTLA-4 were also diminished in this treated population. Similar results were obtained analyzing LN Tregs (*data not shown*). Notably, in contrast to the findings with JQ1, EP11313 treatment did not alter the expression of any of these phenotypic Treg markers (Figure 3).

### EP11313 Regulates Inflammatory Cytokines but Spares the Il-2 Pathway and Effector Molecules in Treg Cells

**Figure 4:**
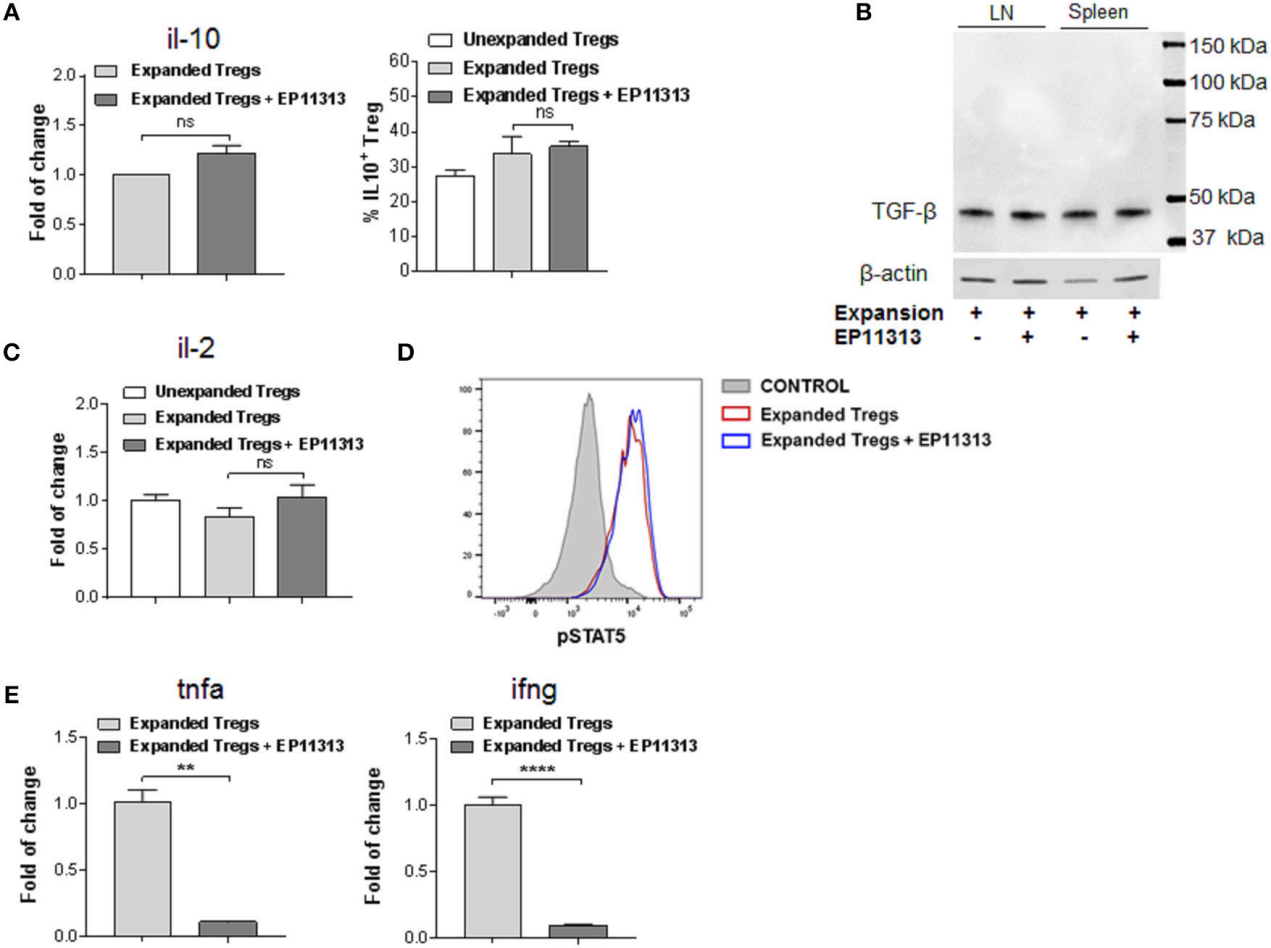
EP11313 regulates inflammatory cytokines but spares the IL-2 pathway and Treg effector molecules. Tregs were expanded with TL1A-Ig + IL-2 in the presence or absence of EP11313 10 mg/kg, mice were sacrificed at day 7 and splenic Tregs (CD4^+^FoxP3^+^) and non-Tregs (CD4^+^FoxP3^−^ and CD4^−^FoxP3^−^) were isolated by FACS **(A–E)**. Quantitative Real-time PCR (qPCR) analysis of il-10 mRNA levels (relative to gapdh) of splenic CD4^+^Foxp3^+^ Tregs sorted from expanded ± EP11313 mice (left). Data are pooled from two independent experiments. IL-10 production by CD4^+^FoxP3^+^ Tregs from LN of expanded and expanded + EP11313 mice after phorbol 12-myristate 13-acetate (1 ng/mL) + ionomycin (1 μM) stimulation for 6 h in the presence of monensin (right) **(A)**. Western Blot analysis of TGF-β levels in sorted Tregs from LN and spleen of mice treated *in vivo* with TL1A-Ig + low dose IL-2 in the presence or absence of EP11313. β-actin was used as a loading control **(B)**. qPCR analysis of il-2 mRNA levels (relative to gapdh) of splenic non-Treg population sorted from unexpanded, expanded or expanded + EP11313 treated mice **(C)**. Representative pSTAT5 staining shown by flow cytometry in CD4^+^FoxP3^+^ Tregs from peripheral blood of expanded ± EP11313 treated B6-FoxP3^RFP^ animals 1 h after final IL-2 injection (3 mice/group) **(D)**. qPCR analysis of tnfa (left) and ifng (right) mRNA levels (relative to gapdh) of splenic non-Treg population sorted from expanded or expanded + EP11313 treated mice **(E)**. Data representative of two independent experiments. ns, not significant. **p < 0.01; ****p < 0.0001.

The above findings demonstrate that EP11313 did not interfere with expansion and phenotype of expanding Treg cells. To examine whether treatment with this BETi altered molecules that mediate Treg function, highly purified CD4^+^FoxP3^+^ Tregs were isolated from animals undergoing expansion in the presence or absence of EP11313 treatment (Figure 4, Figure S1E in Supplementary Material). IL-10 RNA and protein levels were not altered in Tregs exposed to this BETi (Figure 4A). We also did not detect differences in TGF-β protein levels from sorted Treg populations obtained from the spleen and lymph nodes of treated mice (Figure 4B).

Since IL-2 is required for Treg function and survival, we addressed if this cytokine was present in recipients of EP11313 treated mice. Therefore, a Treg negative population (conventional CD4, CD8, NK, macrophage/monocyte and low numbers of contaminating B cells not depleted by sIg treatment) was examined for this cytokine. No differences in the RNA levels of il-2 were identified in these cells from animals undergoing expansion in the presence or absence of this BETi (Figure 4C). Since phosphorylation of STAT5 (pSTAT5) is required for IL-2R signaling, levels of this protein were examined within the Treg populations (Figure 4D). Importantly, no differences were observed in pSTAT5 expression in Tregs undergoing expansion from BETi treated vs. untreated animals (Figure 4D). To validate that EP11313 treatment affected gene transcription in treated animals, RNA from the sorted Treg negative populations was also examined for tnfa and ifng. These inflammatory cytokine RNA were significantly decreased in this population (Figure 4E).

### Recipients Transplanted With Expanded Donor Tregs and Treated With EP11313 Demonstrate Diminished GVHD Early Post-HSCT

**Figure 5:**
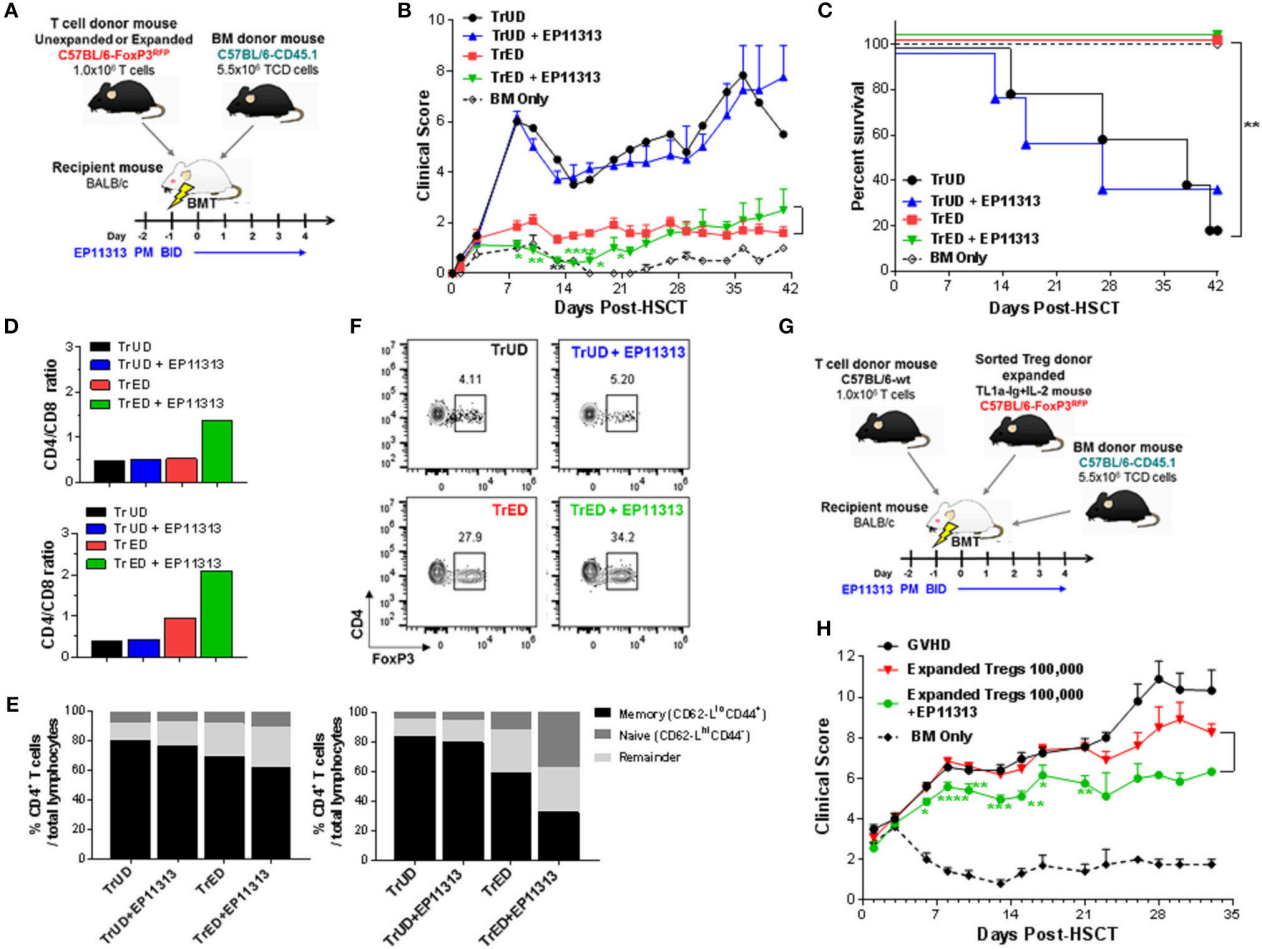
Recipients transplanted with cells from Treg expanded donors (TrED) or low numbers of purified expanded Tregs and treated with EP11313 exhibited diminished acute GVHD after MHC-mismatched aHSCT. An HSCT was performed on Day 0 utilizing a B6 BALB/c donor/recipient mouse model involving a complete MHC mismatch Lethally irradiated (8.5 Gy) BALB/c mice received 5.5 × 10^6^ TCD B6-CD45.1 BM cells and spleen cells from expanded (TL1A-Ig + low dose IL-2 = TrED group) or unexpanded B6-FoxP3^RFP^ (= TrUD group) donor mice adjusted to contain 1.0 × 10^6^ total T cells. EP11313 10 mg/kg or vehicle were given i.p. from day −2 to 4 post-HSCT **(A–F)**. **(A)** Experimental design of the complete MHC-mismatched aHSCT model used in these studies **(A)**. Clinical GVHD scores (0 = no disease and 10 = severe) **(B)** and survival curves **(C)** are presented (*n* = 8 mice/group, *n* = 4 mice/BM Only group). Recipients of TrED and EP11313 demonstrated ameliorated clinical GVHD. *p < 0.05; **p < 0.01; ****p < 0.0001 TrED + EP11313 vs. TrED for clinical score. One week after transplant, the spleens and LNs were evaluated **(D–F)**. Higher CD4/CD8 ratio were found in TrED + EP11313 recipient spleen (top) and LN (bottom) compared to TrED **(D)**. The T cell naïve/memory compartment was analyzed by flow cytometry (CD44/CD62L) in spleen and LN. The naïve compartments of CD4^+^ cells (CD44^−^CD62-L^hi^) in spleen (left) and LN (right) were increased while the T effector/memory (CD44^+^CD62-L^lo^) diminished in TrED + EP11313 recipients compared to TrED treated animals **(E)**. Tissues were pooled from 2 to 3 mice/group **(D,E)**. Representative flow cytometry plots of splenic CD4^+^FoxP3^+^ Treg frequency of the indicated groups are shown **(F)**. A complete MHC-mismatched aHSCT was performed (as in **A**) transplanting sorted CD4^+^FoxP3^+^ expanded Tregs (100,000) together with B6-WT 1 × 10^6^ splenic T cells and 5.5 × 10^6^ TCD B6-CD45.1 BM cells. The experimental design of the complete MHC-mismatched aHSCT model used in these studies **(G)**. Clinical scores of recipient groups showed decreased scores in mice receiving 100,000 expanded donor Tregs + EP11313 **(H)**. Data is representative of two independent experiments (*n* = 8 mice/group). *p < 0.05; **p < 0.01; ***p < 0.001; ****p < 0.0001 Expanded Tregs + EP11313 vs. Expanded Tregs for clinical score.

The above findings support the notion that selected BETi can be combined with Treg cells to prevent GVHD following allogeneic-HSCT. Accordingly, we performed a transplant using a fully MHC-mismatched aHSCT model (B6 → BALB/c) (Figure 5A). Groups of recipients received unfractionated spleen cells (adjusted to contain 1 × 10^6^ T cells) from Treg expanded donors (TrED) or unexpanded donors (TrUD) in the presence or absence of short-term (Day −2 to 4) EP11313 treatment (Figure 5A). Using this protocol, treatment with EP11313 only did not diminish GVHD clinical scoring (Figure 5A). As we have reported, recipients of TrED did exhibit decreased GVHD clinical scores and increased survival compared to recipients receiving TrUD (8) (Figures 5B,C). Interestingly, the combinatorial strategy of TrED plus EP11313 treatment significantly lowered GVHD scores during the first 3 weeks post-HSCT and did not diminish overall (i.e., 100%) survival (Figures 5B,C). To obtain sufficient cell numbers, tissues were pooled from animals 1-week post-HSCT. TrED + EP11313 treatment resulted in increased CD4/CD8 ratios in the spleen and lymph nodes at this time (Figure 5D). More apparent in the lymph node T cells, there was a diminishment of the CD4 Teff/mem (CD44^+^CD62L^lo^) population and an increase in CD4 T naïve (CD44^−^CD62L^hi^) cells from combination (TrED+BETi) treated mice (Figure 5E). As anticipated, recipients of TrED had higher levels of Treg cells compared with recipients of TrUD 1-week post-HSCT in the spleen (Figure 5F). Consistent with the EP11313 and Treg expansion findings above (Figures 1–4), mice receiving the combinatorial (TrED + EP11313) strategy contained similar splenic Treg levels compared with recipients of TrED alone (Figure 5F). In this context, our recent studies reported that <1.75 × 10^5^ two-pathway expanded donor Tregs were not sufficient to ameliorate GVHD in fully mismatched aHSCT recipients (8). Therefore, transplants were performed using 1 × 10^5^ highly purified donor expanded Tregs (Figure S5 in Supplementary Material) alone or in combination with short-term EP11313 treatment of recipients (Figure 5G). The results of two independent pooled transplants demonstrated a significant decrease (up to 3 weeks post-aHSCT) in the clinical GVHD scores between recipients of purified Tregs alone and those receiving the BETi from Days −2 to 4 TrED (Figure 5H). No differences in overall survival between these groups was detected (data not shown).

**Figure 6:**
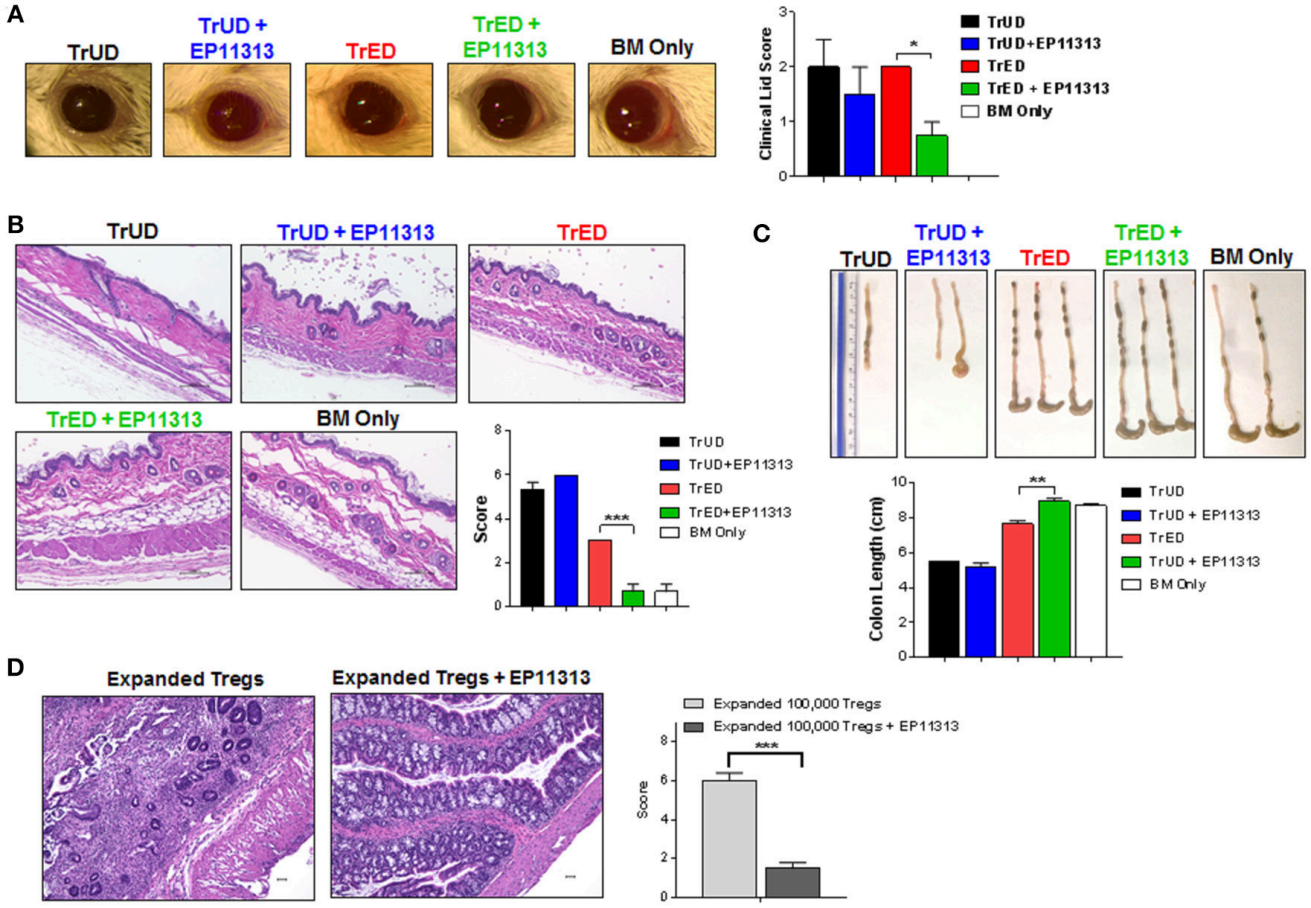
Diminished GVHD in target tissues of recipients treated with EP11313 and TrED. A complete MHC-mismatched aHSCT was performed (as in Figure 5) by transplanting 5.5 × 10^6^ TCD B6-CD45.1 BM cells and spleen cells from expanded (TL1A-Ig + low dose IL-2: TrED group) or unexpanded B6-FoxP3^RFP^ (TrUD) donor mice adjusted to contain 1.0 × 10^6^ total T cells. EP11313 10 mg/kg or vehicle were given i.p. from day −2 to 4 post-HSCT. Four to seven weeks after transplant, **(A)** Representative photographs of clinical ocular differences from the indicated groups (left) and diminished lid scores (right) in TrED + EP11313 treated groups vs. TrED (4 weeks post-transplant) **(A)**. Representative H&E stained sections from skin 5 weeks after aHSCT showed that TrED + EP11313 treatment resulted in more normal architecture with less fibrosis and dermal thickening **(B)**. Pathology scores for these tissues are shown **(B)**. Colon length 7 weeks post-aHSCT was longer in recipients of TrED + EP11313 treatment **(C)**. Representative H&E stained sections from colon 5 weeks after aHSCT of mice treated with 100,000 expanded donor Tregs ± EP11313. Colons from 100,000 expanded Tregs + EP11313 recipients showed mild inflammation and no distortion of the villi compared with colons from 100,000 expanded Tregs alone. Pathology scores for these tissues are shown on the right **(D)**. Magnification 100× for colon and 200× for skin. Values are means ± SEM. *p < 0.05; **p < 0.01; ***p < 0.001.

One and two months post-HSCT, clinical, histological and pathology assessments indicated lower ocular adnexa involvement with less clinical lid edema and closure (Figure 6A) and decreased skin involvement as assessed by overall thickening and fibrosis (Figure 6B). Thymic weight was superior in TrED + EP11313 vs. TrUD + EP11313 and colon length in the former was virtually identical to control BM alone transplanted recipients and significantly greater compared to recipients of TrED alone (Figure S6 in Supplementary Material and Figure 6C, respectively). Moreover, histological assessment of the colon 5 weeks post-aHSCT showed mucosal thickening and severe inflammation with villi distortion in recipients of only 1 × 10^5^ purified expanded Tregs. In contrast, colons from mice receiving 1 × 10^5^ expanded Tregs with EP11313 exhibited a mild inflammation and no disruption of villi architecture (Figure 6D).

## Discussion

The bromodomain and extra-terminal (BET) proteins have a central role in regulating transcription of inflammatory and oncogenic factors and have emerged as attractive druggable targets with therapeutic potential (14, 30). Increasing pre-clinical data, completed and ongoing clinical trials (14) (NCT01943851, NCT01587703, NCT01713582) have demonstrated that BET inhibitors (BETi) possess anti-cancer and anti-inflammatory activity (12, 13, 16, 31). The objective of the present study was to identify a BETi which did not interfere *in vivo* with CD4^+^FoxP3^+^ regulatory T cell (Treg) expansion and function so it could be utilized together with Tregs following aHSCT to ameliorate graft-versus-host disease (GVHD). Notably, *in vitro* analysis of a bromodomain inhibitor of CBP/EP300 reduced human Treg differentiation and suppressive function (18). Interestingly, our studies examining BETi *in vivo* demonstrated that JQ1 interfered with Treg expansion and altered subset distribution and phenotype. In contrast, we found that the BETi EP11313 did not impair the basal (un-manipulated) or the expanded (TL1A + low dose IL-2) Treg compartments. Remarkably, administration of low levels of EP11313 at the time of allogeneic HSCT together with adoptive transfer of expanded Tregs further diminished GVHD.

Regulatory T cells (Tregs) have a critical role in the immune system by maintaining immune homeostasis and preventing occurrence of autoimmune disease (32–34). IL-2 signaling via the high affinity IL-2R (CD25) results in phosphorylation of STAT5 and is necessary for the maintenance and expansion of CD4^+^FoxP3^+^ Tregs (19, 20). Adoptive transfer of regulatory T cells (Tregs) has emerged as a promising therapy for solid organ transplantation, autoimmune diseases and GVHD following aHSCT (3, 4, 6, 35, 36). Our group and others have shown the effectiveness of donor Tregs as a prophylactic strategy to prevent development of GVHD (3–5). We have previously reported that Tregs can be markedly expanded and selectively activated with increased functional capacity by targeting two receptors, i.e., TNFRSF25 and CD25 with TL1A-Ig and low dose IL-2, respectively (7, 8). Additionally, expanded Treg therapy was shown to be as effective as post-transplant cyclophosphamide for GVHD prophylaxis but the former promoted more rapid thymic reconstitution providing earlier recovery of recipient immune function (37). Acute GVHD occurs when donor T cells are primed by recipient antigens subsequently eliciting a rapid inflammatory response (“cytokine storm”) in the host. Because GVHD is promoted by inflammatory cytokines and donor T cells, we reasoned regulating both components is a rational strategy to abrogate onset of this disorder. Significant numbers of Tregs are required to inhibit alloreative T effector cells which induce GVHD. Accordingly, development of a successful combinatorial approach must include a BETi which does not interfere with Treg function or proliferation. During Treg expansion induced by TL1A-Ig + IL-2 stimulation, JQ1 treatment impaired their peripheral frequency and altered several key Treg differentiation and functional molecules including ICOS-1, Nrp-1, KLRG-1 as well as, PD-1 and CTLA-4 which were also reported to be reduced in Tregs by CBP/EP300 bromodomain inhibitors (18). While additional experiments are needed, based on JQ1's capacity to inhibit c-myc, is possible based on our data thus far, that this BETi is affecting more proliferative Treg subsets, i.e., cTregs rather than more differentiated eTregs. Notably, JQ1 has also been found to inhibit frequency and function of tumor infiltrating Tregs in non-small cell lung cancer (38, 39). However, a non-structurally related BETi, EP11313 did not exhibit this pattern of alteration as no effect on frequency was observed in either these intentionally expanded—or homeostatic Treg compartments. It should be noted that although the ½ lives of both BETi are not different the results found that even in the presence of higher amounts of EP11313 (3X) vs. JQ1 there was no reduction in Treg proliferation by the former. These findings were consistent with the observations that IL-2 production by non-Treg cells was not diminished in EP11313 treated animals. Interestingly, Treg pSTAT5 levels were also not diminished after BETi treatment *in vitro* or *in vivo*. This finding contrasts reported observations that JQ1 inhibited STAT5 phosphorylation and transcriptional activity in monocyte/dendritic cells (22). However, similar to our results, it was previously reported that JQ1 or BRD2 downregulation diminishes STAT5 function through phosphorylation-independent mechanisms in lymphocytic leukemias (21). Although we anticipated that due to Treg proliferation differences in mice treated with either JQ1 or EP11313, STAT5 phosphorylation may have been differentially affected by these BETi's. In contrast to other cell populations, it appears BETi STAT5 regulation of lymphoid lineage populations involves a different mechanism. It was previously reported that STATs can be acetylated under certain conditions (40). Thus, it is possible that STAT5 or proteins involved in this signaling pathway in expanding Tregs or hematologic cancers does not contain acetylated lysine residues which are present following LPS stimulation of dendritic cells (21, 22).

Importantly, using a brief, i.e., 1 week protocol of EP11313 10 mg/kg (a low dose of BETi) as mentioned above, several differentiation and functional molecules (ex. ICOS-1, Nrp-1, PD-1) were not altered in Tregs. Moreover, this regimen of EP11313 did not reduce IL-10 and TGF-β–two key Treg suppressive molecules. As the objective of the present studies was to combine the use of BETi with donor Tregs to more effectively regulate GVHD onset, the findings above support the strategy of using both EP11313 together with Tregs. A BETi was reported to inhibit cytokine expression and APC function in dendritic cells and to decrease cytokine secretion and T cell expansion *in vivo* (17). Short-term administration of IBET151 early during BMT reduced GVHD severity supporting the notion that inhibiting BET proteins may serve as an approach for preventing GVHD (17). However, we observed that I-BET151 *in vivo* treatment for 1 week markedly reduced B cell levels in the spleen and therefore, would not be useful in the GVHD setting because it might affect recipients' immune reconstitution (data not shown). Importantly, EP11313 possesses distinct pharmacological properties, for example, this BETi is less extruded by P-glycoprotein (expressed on APC and activated T cells) across the cell membrane in comparison with I-BET762 and I-BET151 (MDR/PgP-MDCK efflux ratio BA/AB: 1.3, 27.9, 12.2, respectively, personal observation). EP11313 is therefore more highly retained intracellularly and this persistence may increase the regulation of inflammatory cytokines. Based on these properties together with the above mentioned findings showing that Tregs are not impaired by EP11313, short-term treatment (day −2 to 4) with expanded Tregs was utilized in an aHSCT. This approach was found to diminish early GVHD clinical scores including decreased ocular and skin involvement. Using highly purified donor TL1A-Ig + low dose IL-2 expanded Tregs, this second and more direct assessment of the combinatorial strategy further supported the notion that selective BETi can be used for treatment in combination with adoptive Treg therapy. Although short-term treatment with EP11313 did not enhance overall survival, BETi utilization in pre-clinical tumor models as well as clinical oncology trials have involved long-term (ex. months) administration of higher BETi doses (up to 50 mg/kg), therefore increasing the duration of BETi low dose treatment post-HSCT may further improve recipient outcomes.

It has been demonstrated that adoptive transfer of Tregs can effectively abrogate GVHD while maintaining graft-versus tumor or leukemia/lymphoma (GVT, GVL) (41, 42). In this context, we previously demonstrated using A20^luc/YFP^ cells (murine B cell lymphoma) that transplanting donor TL1A-Ig + IL-2 spleen cells (containing ~4 × 10^5^ Tregs) GVHD was significantly reduced and GVL was preserved (7). In hematologic malignancies, BETi have demonstrated to possess effective anti-tumor activity by repressing aberrant oncogenic transcription (11, 43, 44). Importantly, here we showed besides GVHD amelioration, a direct effect of BETi EP11313 on A20 ^luc/YFP^ cell survival and proliferation. Examination of other mouse tumor cell lines i.e., EL4 (thymoma) and P815 (mastocytoma) indicated the latter was resistant to BETi effect on cell survival so not all tumors are equally sensitive to these compounds (SC, RBL unpublished observations). Our results indicate that JQ1 and EP11313 have similar anti-cancer effects (IC50 = 0.19 and 0.28 μM, respectively) in A20^luc/YFP^ tumor cells. However, these BETi exhibit significantly different biological effects on Tregs, where EP11313 have no interference with Treg proliferation, phenotype and function. We hypothesize that a strategy using expanded Tregs and EP11313 may not impair GVL and could directly inhibit tumor growth. Thus, the overall mechanism proposed involves Treg mediated suppression of donor allo-reactive T cells, BETi blockage of inflammatory cytokines; and direct BETi anti-tumor activity. In total, we posit that selected BETi treatment together with expanded Treg therapy represents a novel and potentially effective combinatorial strategy for ameliorating hematologic cancer and GVHD.

## Author Contributions

SC designed research studies, discussed data sets, conducted experiments, analyzed data and interpreted data, wrote the manuscript. CL, HB, JM, DS, and BK conducted experiments and analyzed data. IL, DW, and CB performed research, analyzed and interpreted data, and edited the manuscript. SB supported the research. KK discussed Treg studies and supported the research. VLP discussed Treg studies and BETi and supported the research. CW discussed data and manuscript and supported the research. RBL designed research studies, discussed data sets, wrote the paper, supervised and supported the research.

### Conflict of Interest Statement

RBL is a scientific advisory board member of Heat Biologics and a consultant for Heat Biologics and Pelican Therapeutics. The remaining authors declare that the research was conducted in the absence of any commercial or financial relationships that could be construed as a potential conflict of interest.
