# Supplementary material for: BET Bromodomain Inhibitors Which Permit Treg Function Enable a Combinatorial Strategy to Suppress GVHD in Pre-clinical Allogeneic HSCT
Source: Front Immunol. 2019 Jan 24;9:3104. doi: 10.3389/fimmu.2018.03104 (PMC6353853; doi:10.3389/fimmu.2018.03104)
Supplement: Supplementary file 1 [file Data_Sheet_1.docx]

Supplementary Material

**BET Bromodomain Inhibitors Which Permit Treg Function Enable a Combinatorial Strategy to Suppress GVHD**

**in Pre-clinical Allogeneic HSCT**

Sabrina N. Copsel*, Casey O. Lightbourn, Henry Barreras, Ines Lohse, Dietlinde Wolf, Cameron S. Bader, John Manov, Devangi Shah, Shaun P. Brothers, Victor L. Perez, Krishna V. Komanduri, Claes Wahlestedt, Robert B. Levy*

***Correspondence**:

Robert B. Levy, Ph.D.

[rlevy@med.miami.edu](mailto:rlevy@med.miami.edu)

Sabrina Copsel, PhD

[snc49@med.miami.edu](mailto:snc49@med.miami.edu)

SUPPLEMENTARY FIGURES Figure S1

Figure S2


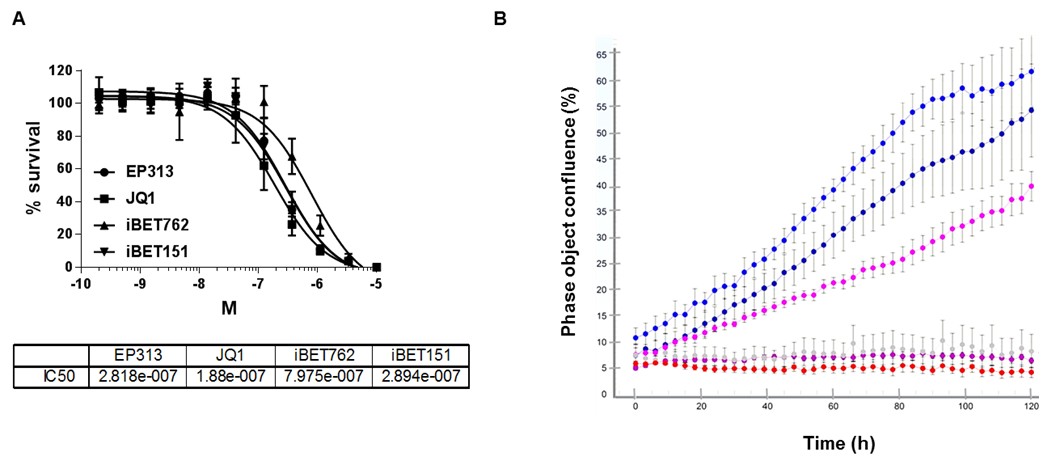


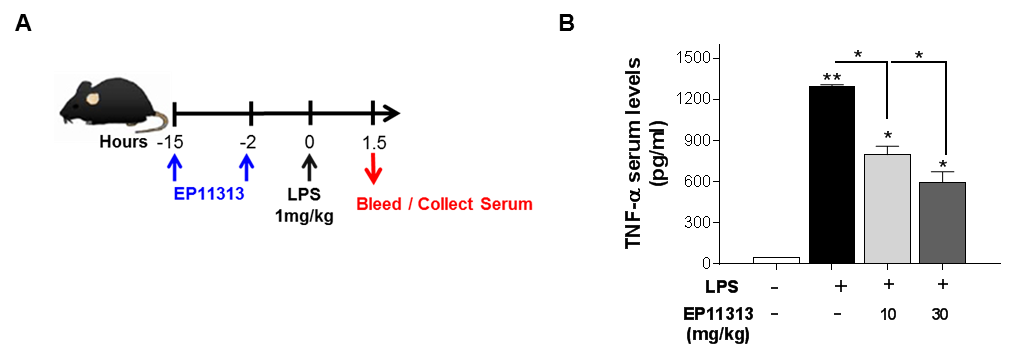
Figure S3

**
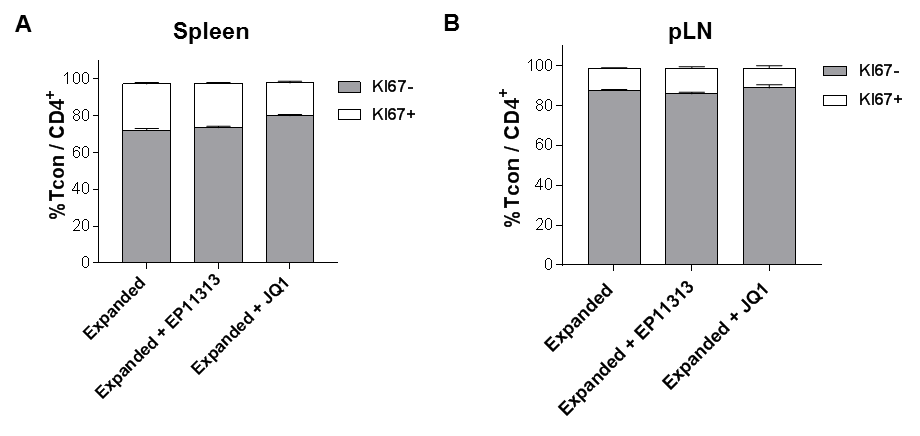
Figure S4**

**
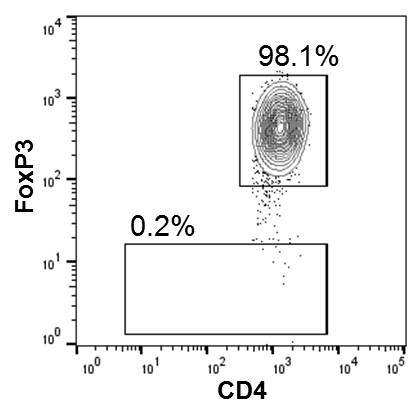
Figure S5**

**Figure S6**

**
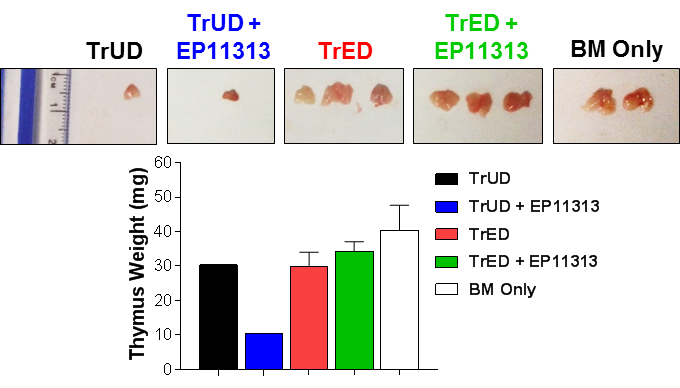
**

**SUPPLEMENTARY FIGURE LEGENDS**

**Figure S1. *In vivo* treatment with BETi EP11313 does not interfere with Treg basal compartment (A-D)** Mice were injected i.p with EP11313 (10 mg/kg) or administered vehicle on days -1 to 6. Mice were sacrificed on day 7. Experimental design of the EP11313 *in vivo* treatment in these set of studies **(A)** CD4^+^ frequency (%) in the spleen and lymph nodes (LN) **(B)** of mice treated with BETi EP11313 or vehicle (control). Treg frequency (%) within the CD4 fraction (CD4^+^FoxP3^+^ / CD4^+^) in the spleen and lymph nodes **(C)**. Sorted Tregs from B6-FoxP3^RFP^ mice treated with EP11313 or vehicle were compared for functional activity using an *in vitro* Treg suppression assay and Tregs from EP11313 treated animals demonstrated no differences compared to control Tregs in suppressive activity on a per cell basis **(D)**. Experimental design showing the *in vivo* treatment with TL1A-Ig (on days 1 to 4) and rmIL-2 bound to the anti-IL-2 mAb (JES6-5H4, on days 4 and 6) and EP11313 (10 mg/kg) or administered vehicle on days -1 to 6. Mice were sacrificed on day 7 **(E)**. Total numbers of Tregs in the peripheral lymph nodes of mice undergoing Treg expansion (TL1A-Ig + low dose IL-2) in the presence or absence of BETi EP11313. Data are representative of 2 independent experiments (n = 2 mice/group) **(F)**. Data are expressed as means ± SEM and were analyzed by a two-tailed unpaired t test. ns = not significant.

**Figure S2.** **BETi reduces lymphoma cell survival and proliferation.** A20 mouse lymphoma cells were seeded in 384-well plates and treated with the BETi in dose response for 72h. A dose-dependent decrease in proliferation was observed in response to all of the tested inhibitors, although IC_50_ vales differ between different compounds **(A)**. Cells were seeded in 24-well plates and treated with EP11313 at IC_50_ in order to evaluate proliferation; (●) vehicle, (●) EP11313 50 nM, (●) EP11313 100 nM, (●) EP11313 500 nM, (●) EP11313 1 μM, (●) EP11313 1.5 μM. A significant and lasting reduction in proliferation was observed in response to treatment **(B)**.

**Figure S3. BETi EP11313 administration reduces TNF-α serum levels.** Mice were injected i.p with EP11313 (10 or 30 mg/kg) or vehicle prior LPS 1 mg/kg treatment. After 1.5 hours mice were bleed. Experimental design is shown **(A).** TNF-α serum levels were significantly decreased after EP11313 10 mg/kg *in vivo* treatment or even higher diminution was observed with 30 mg/kg treatment **(B)**. Data are expressed as means ± SEM and were analyzed by one-way ANOVA. *p<0.05; **p<0.01.

**Figure S4. EP11313 or JQ1 do not inhibit conventional CD4 cell proliferation.** Mice were injected i.p with TL1A-Ig and rmIL-2 bound to anti-IL-2 mAb as in Figure S1 and EP11313 (10 mg/kg), JQ1 (5-10 mg/kg) or vehicle (on days -1 to 6). Mice were sacrificed on day 7. *In vivo* treatment with EP11313 or JQ1 did not alter conventional CD4^+^ FoxP3^-^ T cells frequency (%) of total CD4^+^ cells in the spleen **(A)** and pLN **(B)**. Data are expressed as means ± SEM, n=3 mice/group.

**Figure S5. Purity of sorted expanded Tregs.** Mice were treated with TL1A-Ig and rmIL-2 bound to anti-IL-2 mAb and were sacrifice on day 7. Splenic CD4^+^FoxP3^+^ Tregs were sorted. Representative contour plot showing the purity (>98%) of expanded CD4^+^FoxP3^+^ Tregs after FACS.

**Figure S6. Thymic size and weight of recipients treated with unexpanded or expanded Tregs with EP11313 or vehicle.** A complete MHC-mismatched aHSCT was performed (as in **Figure 5**) by transplanting 5.5x10^6^ TCD B6-CD45.1 BM cells and spleen cells from expanded (TL1A-Ig + low dose IL-2:TrED group) or unexpanded B6-FoxP3^rfp^ (TrUD) donor mice adjusted to contain 1.0 x10^6^ total T cells. EP11313 10 mg/kg or vehicle were given i.p from day -2 to 4 post-HSCT. Seven weeks after transplant, mice were sacrificed. Representative photographs of thymuses (top) and thymic weight (bottom) from the indicated groups. Thymic weight was superior in TrED+EP11313 versus TrUD+EP11313.

**SUPPLEMENTARY MATERIALS AND METHODS**

**Table S1. Antibodies used for flow cytometry**

| **mAbs** | **Clone** |
| --- | --- |
| CD4 | RM4-5 or GK1.5 |
| CD8 | 53-6.7 |
| CD25 | PC61 |
| CD39 | 24DMS1 |
| CD44 | IM7 |
| CD45.1 | A20 |
| CD62L | MEL-14 |
| CD73 | eBioTY/11.8 |
| CD103 | 2E7 |
| CTLA4 | UC10-4B9 |
| FoxP3 | FJK-16s |
| H2K^b^ | AF6-88.5 |
| I-COS | 15F9 |
| KLRG1 | 2F1 |
| Ly-6C | HK1.4 or AL-21 |
| Nrp-1 | 3DS304M |
| PD-1 | 29F.A12 |

**Table S2. Primers sequences used for qPCR**

| **Specific primers** | **Forward** | **Reverse** |
| --- | --- | --- |
| il-2 | 5’-GTGCTCCTTGTCAACAGCG -3’ | 5’- GGGGAGTTTCAGGTTCCTGTA -3’ |
| il-10 | 5’-GCTCTTACTGACTGGCATGAG-3’ | 5’-CGCAGCTCTAGGAGCATGTG-3’ |
| tnf-a | 5’- CTGAACTTCGGGGTGATCGG -3’ | 5’- GGCTTGTCACTCGAATTTTGAGA -3’ |
| ifng | 5’-ATGAACGCTACACACTGCATC-3’ | 5’- CCATCCTTTTGCCAGTTCCTC-3’ |
| gapdh | 5’-AGGTCGGTGTGAACGGATTTG-3’ | 5’- TGTAGACCATGTAGTTGAGGTCA-3’ |
